# Decoding inflammation: first-trimester biomarkers as predictive tools for gestational diabetes

**DOI:** 10.1590/1806-9282.20251962

**Published:** 2026-06-29

**Authors:** Samet Kırat

**Affiliations:** 1 Kafkas University, Faculty of Medicine, Department of Gynecology and Obstetrics – Kars, Turkey.

**Keywords:** Blood platelets, Diabetes mellitus, Inflammation, Lymphocytes, Neutrophils

## Abstract

**OBJECTIVE:**

Gestational diabetes mellitus is a pregnancy complication associated with adverse maternal and neonatal outcomes. The aim of this study was to evaluate the hemoglobin-albumin-lymphocyte-thrombocyte, Aggregate Systemic Inflammation Index, Systemic Inflammatory Response Index, Systemic Immune-Inflammation Index, neutrophil-lymphocyte ratio, platelet-lymphocyte ratio, and monocyte-lymphocyte ratio measured in the first trimester as early predictive biomarkers for gestational diabetes mellitus.

**METHODS:**

This retrospective cohort study included 588 pregnant women: 294 women with gestational diabetes mellitus and 294 matched controls. Demographic, obstetric, and first-trimester hematologic data were collected.

**RESULTS:**

Systemic Immune-Inflammation Index (p<0.001), neutrophil-lymphocyte ratio (p<0.001), and platelet-lymphocyte ratio (p<0.001) levels were significantly higher in those with gestational diabetes mellitus, while hemoglobin-albumin-lymphocyte-thrombocyte (p=0.001) and Aggregate Systemic Inflammation Index (p=0.020) levels were significantly lower than in those without gestational diabetes mellitus. Platelet-lymphocyte ratio (area under the curve=0.679; sensitivity 62.9%, specificity 63.3%) emerged as the strongest inflammatory marker for predicting gestational diabetes mellitus. Neutrophil-lymphocyte ratio (area under the curve=0.645) and Systemic Immune-Inflammation Index (area under the curve=0.610) demonstrated moderate discriminatory ability, whereas hemoglobin-albumin-lymphocyte-thrombocyte (area under the curve=0.422) and Aggregate Systemic Inflammation Index (area under the curve=0.444) showed weak diagnostic performance for gestational diabetes mellitus. In contrast, Systemic Inflammatory Response Index (area under the curve=0.466) and monocyte-lymphocyte ratio (area under the curve=0.475) were not significantly different between groups and lacked clinical relevance.

**CONCLUSION:**

Platelet-lymphocyte ratio, neutrophil-lymphocyte ratio, and Systemic Immune-Inflammation Index are promising biomarkers for early gestational diabetes mellitus prediction in the first trimester. These findings support the role of inflammation in gestational diabetes mellitus pathophysiology and highlight the value of routine hematologic markers in risk stratification.

## INTRODUCTION

Gestational diabetes mellitus (GDM) affects 5–20% of pregnancies and is characterized by glucose intolerance that disappears after delivery, leading to complications for mother and fetus^1^. Women with GDM have increased risks of preeclampsia, cesarean delivery, type 2 diabetes mellitus (DM) postpartum, fetal macrosomia, neonatal hypoglycemia, and later metabolic syndrome. Early diagnosis and management of GDM are critical for maternal-fetal health outcomes^2^.

GDM occurs when the pancreas fails to mount adequate compensation for physiological insulin resistance during pregnancy. Research shows this process may have both metabolic and inflammatory bases^3^. Physiological inflammation during pregnancy with immune system modulation may become uncontrolled and trigger pathological processes^4^. Inflammation increases insulin resistance by disrupting insulin signaling pathways through cytokine release and neutrophil activation^5^. This highlights inflammatory markers’ importance in GDM development and their clinical value for diagnosis and prediction^6^.

The Systemic Immune-Inflammation Index (SII), Systemic Inflammatory Response Index (SIRI), Aggregate Index of Systemic Inflammation (AISI), neutrophil/lymphocyte ratio (NLR), and platelet/lymphocyte ratio (PLR) have been shown to provide meaningful diagnostic information for risk classification and prognosis prediction in cancer, cardiovascular, metabolic, and infectious diseases^7,8^. Additionally, NLR, PLR, monocyte/lymphocyte ratio (MLR), SII, and SIRI have been reported to be higher in the GDM group^9,10^. These findings suggest that systemic inflammation indices may also be associated with GDM and have predictive value in the pre-diagnostic period^9^.

The aim of this study was to evaluate hematologic indices such as hemoglobin-albumin-lymphocyte-platelet (HALP),AISI, SIRI, SII, NLR, PLR, and MLR, which indicate systemic inflammation, to predict GDM. Measuring these indices in early pregnancy to identify GDM risk may enable early intervention and reduce unnecessary oral glucose tolerance tests (OGTT). These findings will determine whether inflammation-based biomarkers are practical and cost-effective for GDM screening.

## METHODS

### Study design

This retrospective study included 588 women who fulfilled the inclusion criteria and presented to the obstetrics and gynecology outpatient clinic of a tertiary university hospital between 2018 and 2025.

### Inclusion criteria

The study included women between the ages of 18 and 55 years, who presented for routine antenatal follow-up, had a singleton pregnancy, and whose medical records were fully accessible from both the hospital information management system and physical archive files.

### Exclusion criteria

Women with known comorbidities, active urinary tract and/or genital tract infections, pregnancies conceived by assisted reproductive techniques, and multiple gestations were excluded.

### Covariates and descriptive data

The demographic and health-related characteristics analyzed were age, number of gravida and parity, employment status, educational level (primary, secondary, or higher education), yearly income level (low, medium, or high), and smoking. The hemogram results of the pregnant women in the first trimester were analyzed, and neutrophil, lymphocyte, monocyte, platelet, hemoglobin, and albumin values were recorded.

### Group selection

GDM diagnosis is based on results from a 75-g OGTT performed between 24 and 28 weeks of gestation according to International Association of Diabetes and Pregnancy Study Groups criteria^11^. The study group comprised pregnant women diagnosed with GDM (n=294), while the control group was selected by random sampling among non-GDM pregnant women in the same period who were followed under similar clinical conditions (n=294) to ensure one-to-one matching.

### Statistical analysis

Data evaluations were performed using Statistical Package for the Social Sciences (SPSS) for Windows (version 26.0; SPSS Inc., Chicago, IL, USA). Numerical data are expressed as medians (minimum–maximum), and categorical data are summarized using frequency and percentage. The assumption of a normal distribution was evaluated with the Kolmogorov– Smirnov test; comparisons between groups in which the data were found to be nonnormally distributed were made with the Mann–Whitney U test. Categorical variables were analyzed using the chi-square (χ^2^) test.

Stepwise linear regression analysis determined factors associated with HALP, AISI, SIRI, SII, NLR, PLR, and MLR scores. First, univariate linear regression analyses were performed for each dependent variable to identify significant variables. Second, only significant variables were included in the multivariate linear regression model for final analysis. Univariate and multivariate logistic regression analyses determined factors affecting GDM risk. Receiver operating characteristic (ROC) curves determined the diagnostic accuracy of markers. Statistical significance was set at p<0.05.

### Ethics approval and consent to participate

This study was approved by the Non-Interventional Clinical Research Ethics Committee of the Kafkas University Faculty of Medicine (26/03/2025, 80576354-050-99/633). This study complied with the recommendations of the Declaration of Helsinki for human biomedical research.

## RESULTS

### Demographic data, obstetric data, and inflammation indices of the total cohort

This study included a total of 588 patients, 294 with GDM and 294 without GDM. The median age at diagnosis was 32 (19–53) years. The median gravida, the median parity, and the median abortion were 2 (1–6), 1 (0–4), and 0 (0–3), respectively. The rate of smokers was 17.9% (n=105), and the rate of employed individuals was 50% (n=294). The educational status was as follows: high school (n=447, 76%), college/university (n=74, 12.6%), and primary school (n=67, 11.4%). The yearly income level was as follows: medium (n=452, 76.9%), low (n=79, 13.4%), and high (n=57, 9.7%). The median values of inflammation indices in the entire cohort were as follows: HALP 0.40 (0.05–6.05), AISI 328.5 (5.26–5981), SIRI 1.54 (0.1–26.5), SII 711.04 (17.5–4398), NLR 3.2 (0.78–24.8), PLR 123.5 (4.5–655.5), and MLR 0.23 (0.03–2.19).

### Comparison of patients with and without gestational diabetes mellitus

SII levels (814.09 [17.55–4398] vs. 661.51 [248.1–2172]; p<0.001), NLR (3.72 [0.78–24.85] vs. 2.82 [0.83–8.10]; p<0.001), and PLR (142.17 [4.5–655.5] vs. 113.25 [46.81–510]; p<0.001) levels were significantly higher in patients with GDM than in those without GDM, whereas HALP (0.37 [0.05–6.05] vs. 0.42 [0.09–1.18]; p=0.001) and AISI (321.45 [5.26–5981] vs. 346.88 [74.63–1347]; p=0.020) levels were lower.

There were no significant differences between the two groups in terms of parity, smoking, employment status, college/university, high yearly income level, SIRI, and MLR levels (p>0.05) (Table 1).

**Table 1:** Comparison of patients with and without gestational diabetes mellitus.

Variables	Group with GDM (n=294)	Group without GDM (n=294)	p-value
Age (years) (median [min–max])	31.5 (19–44)	33 (23–53)	**0.001**
Gravida (median [min–max])	2 (1–6)	2 (1–6)	**<0.001**
Parity (median [min–max])	1 (0–3)	1 (0–4)	0.096
Abortus (median [min–max])	0 (0–3)	0 (0–3)	**<0.001**
Smoking (n, %)	54 (18.4%)	51 (17.3%)	0.747
Employment status (n, %)	154 (52.4%)	140 (47.6%)	0.248
Education (n, %)			
Primary school	44 (15%)	23 (7.8%)	**0.006**
High school	210 (71.4%)	237 (80.6%)	**0.009**
College/university	40 (13.6%)	34 (11.6%)	0.456
Yearly income level (n, %)			
Low	65 (22.1%)	14 (4.8%)	**<0.001**
Medium	198 (67.3%)	254 (86.4%)	**<0.001**
High	31 (10.5%)	26 (8.8%)	0.486
Inflammation indices			
HALP (median [min–max])	0.37 (0.05–6.05)	0.42 (0.09–1.18)	**0.001**
AISI (median [min–max])	321.45 (5.26–5,981)	346.88 (74.63–1,347)	**0.020**
SIRI (median [min–max])	1.53 (0.10–26.58)	1.63 (0.33–5.33)	0.153
SII (median [min–max])	814.09 (17.55–4,398)	661.51 (248.1–2,172)	**<0.001**
NLR (Median [min–max])	3.72 (0.78–24.85)	2.82 (0.83–8.10)	**<0.001**
PLR (Median [min–max])	142.17 (4.5–655.5)	113.25 (46.81–510)	**<0.001**
MLR (Median [min–max])	0.22 (0.03–2.19)	0.25 (0.11–0.80)	0.280

AISI: Aggregate Index of Systemic Inflammation, GDM: gestational diabetes mellitus, HALP: hemoglobin-albumin-lymphocyte-platelet, MLR: monocytelymphocyte ratio, NLR: neutrophil-lymphocyte ratio, PLR: platelet-lymphocyte ratio, SII: Systemic Immune-Inflammation Index, SIRI: Systemic Inflammatory Response Index. Bold values indicate statistically significant results (p<0.05).

### Comparison of gestational diabetes mellitus patients with and without insulin treatment

There was no statistically significant difference in HALP, AISI, SIRI, SII, NLR, PLR, and MLR levels between GDM patients with and without insulin treatment (p>0.05) (Table 2).

**Table 2:** Comparison of gestational diabetes mellitus patients with and without insulin treatment.

Variables	GDM patients with insulin treatment (n=48)	GDM patients without insulin treatment (n=246)	p-value
Age (years) (median [min–max])	31 (19–39)	32 (23–44)	0.102
Gravida (median [min–max])	2 (1–5)	2 (1–6)	0.256
Parity (median [min–max])	1 (0–3)	1 (0–3)	**0.013**
Abortus (median [min–max])	0 (0–2)	0 (0–3)	**0.016**
Smoking (n, %)	15 (31.3%)	39 (15.9%)	**0.021**
Employment status (n, %)	21 (43.8%)	133 (54.1%)	0.250
Education (n, %)			
Primary school	1 (2.1%)	43 (17.5%)	0.074
High school	41 (85.4%)	169 (68.7%)	0.202
College/university	6 (12.5%)	34 (13.8%)	1.0
Yearly income level (n, %)			
Low	13 (27.1%)	52 (21.1%)	0.257
Medium	32 (66.7%)	166 (67.5%)	0.172
High	3 (6.3%)	28 (11.4%)	0.401
Inflammation indices (median [min–max])			
HALP (median [min–max])	0.37 (0.05–0.92)	0.37 (0.09–6.05)	0.516
AISI (median [min–max])	323.75 (64.19–2,316)	321.31 (5.26–5,981)	0.537
SIRI (median [min–max])	1.36 (0.31–7.41)	1.54 (0.10–26.58)	0.750
SII (median [min–max])	835.09 (256.77–3,309)	810.82 (17.55–4,398)	0.461
NLR (median [min–max])	3.72 (1.16–10)	3.72 (0.78–24.85)	0.450
PLR (Median [min–max])	143.47 (70.41–275.55)	142.17 (4.5–655.5)	0.686
MLR (Median [min–max])	0.22 (0.09–0.72)	0.23 (0.03–2.19)	0.891

AISI: Aggregate Index of Systemic Inflammation, GDM: gestational diabetes mellitus, HALP: hemoglobin-albumin-lymphocyte-platelet, MLR: monocytelymphocyte ratio, NLR: neutrophil-lymphocyte ratio, PLR: platelet-lymphocyte ratio, SII: Systemic Immune-Inflammation Index, SIRI: Systemic Inflammatory Response Index. Bold values indicate statistically significant results (p<0.05).

### Receiver operating characteristic analysis for hemoglobin-albumin-lymphocyte-thrombocyte, Aggregate Systemic Inflammation Index, Systemic Inflammatory Response Index, Systemic Immune-Inflammation Index, neutrophil-lymphocyte ratio, plateletlymphocyte ratio, and monocyte-lymphocyte ratio in predicting gestational diabetes mellitus

**Figure 1:**
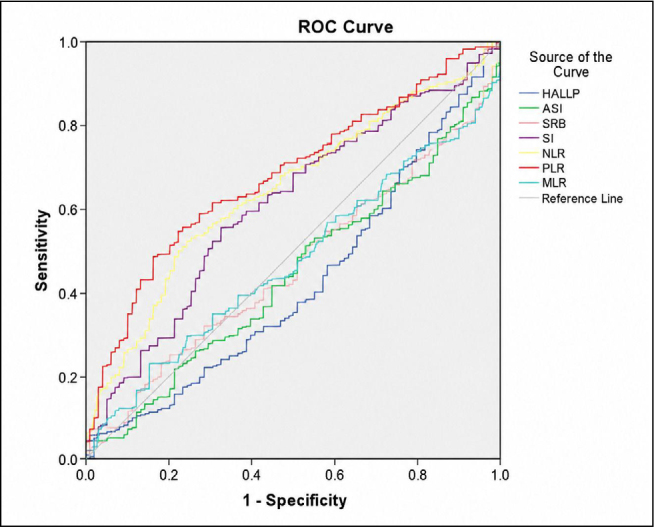
Receiver operating characteristic curve for hemoglobin-albumin-lymphocyte-thrombocyte, Aggregate Systemic Inflammation Index, Systemic Inflammatory Response Index, Systemic Immune-Inflammation Index, neutrophil-lymphocyte ratio, platelet-lymphocyte ratio, and monocyte-lymphocyte ratio in predicting gestational diabetes mellitus.

PLR emerged as the strongest inflammatory marker for predicting GDM (area under the curve [AUC]=0.679; 95%CI 0.636–0.722; p<0.001). The optimal cut-off value was 124.05, which yielded a sensitivity of 62.9% and a specificity of 63.3% (Figure 1). NLR (AUC=0.645; 95%CI 0.601–0.690; p<0.001) and SII (AUC=0.610; 95%CI 0.564–0.655; p<0.001) also showed moderate discriminative performance for GDM. On the other hand, HALP (AUC=0.422; 95%CI 0.376– 0.468; p=0.001) and AISI (AUC=0.444; 95%CI 0.398–0.491; p=0.020) had weak predictive power for GDM. In subgroup analyses by age, in the ≥35 age group, NLR (AUC=0.695; 95%CI 0.627–0.763; p<0.001) in the ≥35 age group and PLR (AUC=0.717; 95%CI 0.663–0.771; p<0.001) in the <35 age group showed the highest discriminatory power in predicting GDM (Table 3).

**Table 3:** Receiver operating characteristic analysis for gestational diabetes mellitus.

Variables	AUC	SE	p	OR (95%CI)	Cutt-off	Sensitivity	Specificity
HALP	0.422	0.024	**0.001**	0.376–0.468	0.40	42.9%	42.9%
AISI	0.444	0.024	**0.020**	0.398–0.491	327.70	48.3%	48%
SIRI	0.466	0.024	0.153	0.419–0.513	1.53	49.3%	49%
SII	0.610	0.023	**<0.001**	0.564–0.655	709.94	59.5%	59.2%
NLR	0.645	0.023	**<0.001**	0.601–0.690	3.20	61.9%	61.2%
PLR	0.679	0.022	**<0.001**	0.636–0.722	124.05	62.9%	63.3%
MLR	0.475	0.024	0.285	0.428–0.521	0.23	47.6%	49%
Age-stratified ROC analysis for GDM							
≥35 years							
HALP	0.393	0.037	0.005	0.319–0.466	0.39	40.8%	41.2%
AISI	0.404	0.037	**0.012**	0.331–0.477	355.20	44.6%	44.1%
SIRI	0.454	0.038	0.234	0.380–0.529	1.63	43.1%	44.1%
SII	0.595	0.037	**0.013**	0.522–0.668	702.90	57.7%	58.8%
NLR	0.695	0.035	**<0.001**	0.627–0.763	3.19	64.6%	64.7%
PLR	0.624	0.036	**0.001**	0.553–0.696	123.01	56.9%	55.9%
MLR	0.465	0.038	0.366	0.391–0.540	0.23	46.2%	47.1%
<35 years							
HALP	0.444	0.031	0.066	0.383–0.504	0.40	43.9%	43.8%
AISI	0.461	0.031	0.204	0.400–0.522	320.05	49.4%	50.0%
SIRI	0.465	0.031	0.261	0.404–0.527	1.44	48.2%	48.4%
SII	0.621	0.030	**<0.001**	0.563–0.680	739.22	61.6%	60.9%
NLR	0.623	0.030	**<0.001**	0.564–0.682	3.16	59.8%	59.4%
PLR	0.717	0.028	**<0.001**	0.663–0.771	127.09	67.1%	67.2%
MLR	0.482	0.032	0.558	0.420–0.544	0.23	48.8%	48.4%

AISI: Aggregate Index of Systemic Inflammation, AUC: area under the curve, GDM: gestational diabetes mellitus, HALP: hemoglobin-albumin-lymphocyte-platelet, MLR: monocyte-lymphocyte ratio, NLR: neutrophil-lymphocyte ratio, PLR: platelet-lymphocyte ratio, SE: standard error, SII: systemic immune-inflammation index, SIRI: Systemic Inflammatory Response Index; CI: confidence interval; OR: odds ratio; ROC: receiver operating characteristic. Bold values indicate statistically significant results (p<0.05).

## DISCUSSION

In this study, we examined the prognostic value of HALP, AISI, SIRI, SII, NLR, PLR, and MLR in predicting GDM. According to our findings, SII, NLR, and PLR levels were higher in patients with GDM, while HALP and AISI levels were lower. According to the results of the ROC analysis, PLR showed the highest diagnostic accuracy in differentiating GDM. NLR and SII had intermediate discriminatory powers, supporting the association between GDM and inflammation. In contrast, HALP and AISI offer limited diagnostic value and are inadequate for clinical use.

PLR is a potential inflammatory biomarker for predicting GDM. Studies show PLR is associated with GDM development and may help identify high-risk pregnant women early^12,13^. However, some studies present conflicting results regarding PLR’s discriminatory power^14^. Meta-analyses show PLR is higher in the GDM group, but suggest interpreting findings cautiously due to study heterogeneity^15^. In our study, PLR was significantly higher in GDM patients (p<0.001). ROC analysis determined PLR as the parameter with the highest discriminative power in predicting GDM. These findings support PLR’s potential utility as a non-invasive, accessible marker for early GDM prediction, particularly alongside other inflammatory indices.

NLR levels are higher in women with GDM in the first and third trimesters and predict GDM development^16^. First-trimester NLR showed a strong association with GDM,with 97.4% diagnostic accuracy^17^. A meta-analysis found NLR levels were higher in GDM patients, independent of ethnicity, diagnostic criteria, and body mass index^15^. High first-trimester SII levels increased GDM risk, being 1.39 times higher in the highest quartile^18^. SII levels were higher in GDM groups, with 66% sensitivity and 65% specificity at cut-off 875^9^, 80.2% sensitivity at 655.75^13^, and discrimination at 607.32^19^. In our study, NLR and SII levels were higher in GDM patients (p<0.001). ROC analysis showed NLR and SII had moderate performance in predicting GDM. These findings suggest NLR and SII may be useful biomarkers for GDM detection, requiring interpretation with other diagnostic parameters.

AISI, a biomarker of systemic inflammation, has shown prognostic value in predicting adverse obstetric outcomes in pregnant women with ulcerative colitis^20^, determining peripheral arterial disease risk in type 2 DM^21^, cardiovascular mortality in hypertensive individuals^22^, and neonatal intensive care unit duration in inflammatory complications like preterm premature rupture of membranes and chorioamnionitis^23^. Low HALP scores were linked to nephropathy, retinopathy, poor stroke prognosis, and mortality in type 2 DM, correlating negatively with HbA1c and glucose levels^24^. AISI and HALP levels have prognostic value in inflammatory conditions, but no studies have examined their use in predicting GDM. In our study, AISI (p=0.020) and HALP (p=0.001) were lower in GDM patients. While AISI and HALP were significant in ROC analysis, their diagnostic power was limited. The lower AISI and HALP levels suggest this index may inadequately reflect GDM’s inflammatory response.

High SIRI levels, especially in the first trimester, have been shown to be significantly associated with the development of GDM^9,18^; however, their ability to predict the need for insulin therapy is limited^13^. High MLR levels have been reported to increase the risk of GDM in the early second trimester and to potentially reflect disease severity, particularly in relation to diabetes complications such as diabetic retinopathy^25^. In contrast, our study found no significant difference in SIRI (p=0.153) and MLR (p=0.280) levels between the GDM and non-GDM groups. This difference may be related to variations in the risk profile of the patient populations in the studies and the gestational weeks at which inflammation was assessed; as our study was conducted in the first trimester and in a relatively lower-risk, homogeneous group, whereas previous studies often included later gestational weeks or higher-risk populations.

This study contributes to the literature by presenting a large-sample analysis evaluating systemic inflammation indices to predict GDM development in the first trimester. The high discriminatory power of PLR suggests its potential as a non-invasive, accessible, and cost-effective early biomarker. However, the retrospective design and single-center nature of the study are the main limitations that restrict the generalizability of findings.

## CONCLUSION

This study demonstrated that PLR, NLR, and SII measured in the first trimester showed significant associations with the development of GDM and are prominent inflammation-based markers in early risk classification. The fact that other indices evaluated under the same clinical conditions provide more limited information suggests that inflammation-based biomarkers should be selectively considered in early pregnancy. These findings support the potential role of markers obtained from routine blood counts in early risk assessment for GDM, and further studies are needed to clarify the place of this approach in clinical practice.

## Data Availability

The datasets generated and/or analyzed during the current study are available from the corresponding author upon reasonable request.
